# A highly generalized classifier for osteoporosis radiography based on multiscale fractal, lacunarity, and entropy distributions

**DOI:** 10.3389/fbioe.2023.1054991

**Published:** 2023-05-19

**Authors:** Jingnan Cui, Cheng Lei Liu, Rachid Jennane, Songtao Ai, Kerong Dai, Tsung-Yuan Tsai

**Affiliations:** ^1^ School of Biomedical Engineering, Shanghai Jiao Tong University, Shanghai, China; ^2^ Department of Radiology, Shanghai Ninth People’s Hospital, Shanghai Jiao Tong University School of Medicine, Shanghai, China; ^3^ IDP Institute, UMR CNRS 7013, University of Orléans, Orléans, France; ^4^ Department of Orthopaedic Surgery, Shanghai Ninth People’s Hospital, Shanghai Jiaotong University School of Medicine, Shanghai, China

**Keywords:** osteoporosis, trabecular degeneration, fractal dimension, entropy, lacunarity, neighborhood component analysis, image dehazing

## Abstract

**Background:** Osteoporosis is a common degenerative disease with high incidence among aging populations. However, in regular radiographic diagnostics, asymptomatic osteoporosis is often overlooked and does not include tests for bone mineral density or bone trabecular condition. Therefore, we proposed a highly generalized classifier for osteoporosis radiography based on the multiscale fractal, lacunarity, and entropy distributions.

**Methods:** We collected a total of 104 radiographs (92 for training and 12 for testing) of lumbar spine L4 and divided them into three groups (normal, osteopenia, and osteoporosis). In parallel, 174 radiographs (116 for training and 58 for testing) of calcaneus from health and osteoporotic fracture groups were collected. The texture feature data of all the radiographs were pulled out and analyzed. The Davies–Bouldin index was applied to optimize hyperparameters of feature counting. Neighborhood component analysis was performed to reduce feature dimension and increase generalization. A support vector machine classifier was trained with only the most effective six features for each binary classification scenario. The accuracy and sensitivity performance were estimated by calculating the area under the curve.

**Results:** Interpretable feature trends of osteoporotic pathological changes were depicted. On the spine test dataset, the accuracy and sensitivity of binary classifiers were 0.851 (95% CI: 0.730–0.922), 0.813 (95% CI: 0.718–0.878), and 0.936 (95% CI: 0.826–1) for osteoporosis diagnosis; 0.721 (95% CI: 0.578–0.824), 0.675 (95% CI: 0.563–0.772), and 0.774 (95% CI: 0.635–0.878) for osteopenia diagnosis; and 0.935 (95% CI: 0.830–0.968), 0.928 (95% CI: 0.863–0.963), and 0.910 (95% CI: 0.746–1) for osteoporosis diagnosis from osteopenia. On the calcaneus test dataset, they were 0.767 (95% CI: 0.629–0.879), 0.672 (95% CI: 0.545–0.793), and 0.790 (95% CI: 0.621–0.923) for osteoporosis diagnosis.

**Conclusion:** This method showed the capacity of resisting disturbance on lateral spine radiographs and high generalization on the calcaneus dataset. Pixel-wise texture features not only helped to understand osteoporosis on radiographs better but also shed new light on computer-aided osteopenia and osteoporosis diagnosis.

## 1 Introduction

Nearly 50% of women and 20% of men suffer from osteoporotic fractures in their lifetime (Office of the Surgeon General (US), 2004; Yamamoto et al., 2020). Asymptomatic osteoporosis often remains overlooked or misdiagnosed until a low-trauma trunk fracture occurs, which leads to hospitalization (Tu et al., 2018). A total of 8.9 million fractures inflicted by osteoporosis occur worldwide each year, causing a significant burden on social health and the economy (Johnell and Kanis, 2006). The widely accepted method of screening for osteoporosis, which involves assessing bone mineral density (BMD) through dual-energy X-ray absorptiometry (DXA), is unable to reach the majority of the high-risk population. This is not only due to DXA’s low availability and cost but also the concern of the X-ray dosage and the low screening rate of <10% in the US (Amarnath et al., 2015). While in China, only 4.3% of women aged ≥50 years have undergone testing, even less women in rural areas are subjected to test, accounting for only 1.9% (National Health Commission of China, Epidemiological investigation of osteoporosis in China, 2018). In addition, acute respiratory distress syndrome such as COVID-19 and glucocorticoid therapy could lead to osteoporosis directly and indirectly (Tang, 2022). Overall, there is a need for an easy-to-use screening tool for osteoporosis.

With the advance in machine learning (ML) (Hassouni et al., 2017), especially deep learning (DL) (Areeckal et al., 2018a; Tecle et al., 2020; Yamamoto et al., 2020; Zhang et al., 2020), radiographic images can be used to diagnose osteoporosis, whereas the multilayer architecture of DL models result in several shortcomings. First, the interpretability of a model is expected. Whether we can replace physician intelligence with a DL model trained from a labeled dataset largely depends on its interpretability for clinicians (Smets et al., 2021). Nevertheless, the state-of-the-art DL studies lack solid clinical implications (Smets et al., 2021). Second, the reliability of multilayer DL models relies on the quantity and the quality of the whole labeled dataset. For complex DL model training, thousands of well-defined images are commonly required to avoid overfitting and inconsistent prediction (Scanlan et al., 2018; Xiao et al., 2020). In practice, diagnosing and disclosing the essential difference between health and osteoporotic bone trabecular structure on a limited sample size is a big challenge.

Indeed, diagnostic reasoning is derived from the first principle (Reiter, 1987). According to pathophysiology, the osteoporotic fracture risk is aroused by the loss of bone tissue (Cosman et al., 2014; Smets et al., 2021). From the microarchitectural point of view, individual 3D trabecular plates of bone are lost as a microscopic representation of bone loss, leaving an architecturally weakened structure with significantly reduced mass (Smets et al., 2021). Referring to histomorphometry, the 3D trabecular bone structure changes correlate with the 2D fractal descriptor of their projections (Scanlan et al., 2018). Further research has demonstrated that the self-similarity parameter of the 3D fractal character holds an influencing factor of 0.5 on its 2D projection. In prior studies (Jennane et al., 2001; Yger, 2014; Palanivel et al., 2020), textual features such as fractal or lacunarity inferred from histomorphometry were successfully accounted for osteoporosis. However, most of them focused on homogeneous features such as fractal dimension measured by fractal Brownian motion (fBm) (Harrar et al., 2013; Hassouni et al., 2017), which could not describe a heterogeneous texture pattern. Studies on microarchitecture have shown the episodic nature of osteoporotic lesions (Greenwood et al., 2018; Hussain and Han, 2019). The subtle trabecular architectural changes induce a negligible difference in spatial dispersion patterns, especially in the osteopenia stage (Jennane et al., 2001). Therefore, an interpretable and pixel-level detection method can be a reliable tool to identify osteoporotic characterization on radiographs at an early stage, providing a better understanding of the pathological process of osteoporosis *via* texture features. Thus, to the best of our knowledge, no work has been devoted to pairwise feature comparison and classification across normal, osteopenia, and osteoporosis on spine X-ray images.

In this study, we analyzed clinical lateral vertebral lumbar spine radiographs. The whole soft tissue around the tummy, intestinal gas, and even the patient’s waist belt are all superimposed in spine radiographs. In parallel, calcaneus radiographs from the textural characterization of a bone (TCB) challenge dataset were also investigated since these images have a clean bone texture with less routine clinical exam coverage. To distinguish osteoporotic radiographs from healthy ones in two different lesion sites, we customized the image enhancement process for those special artifacts and synthesized multiscale pixel-wise fractal distribution, multiscale pixel-wise entropy distribution, and global lacunarity. The six most influential features out of 11 were selected to train support vector machine (SVM) classifiers to achieve high generalization, which could differentiate normal, osteopenia, and osteoporosis cases. In the process of classifier construction, we aimed to

i) Interpret pixel-wise feature trends to better understand osteoporosis’s pathogenesis and train a feature-based SVM classifier for each osteoporotic binary classification scenario
ii) Evaluate the classification performance on a triple-class problem, normal, osteopenia, and osteoporosis, for the clinical dataset, and a binary class problem for the TCB dataset
iii) Compare the proposed classifier to the other two kinds of models, the other feature-based classifiers in the TCB ranking, and the latest DL-based classifiers

## 2 Methodology

**FIGURE 1:**
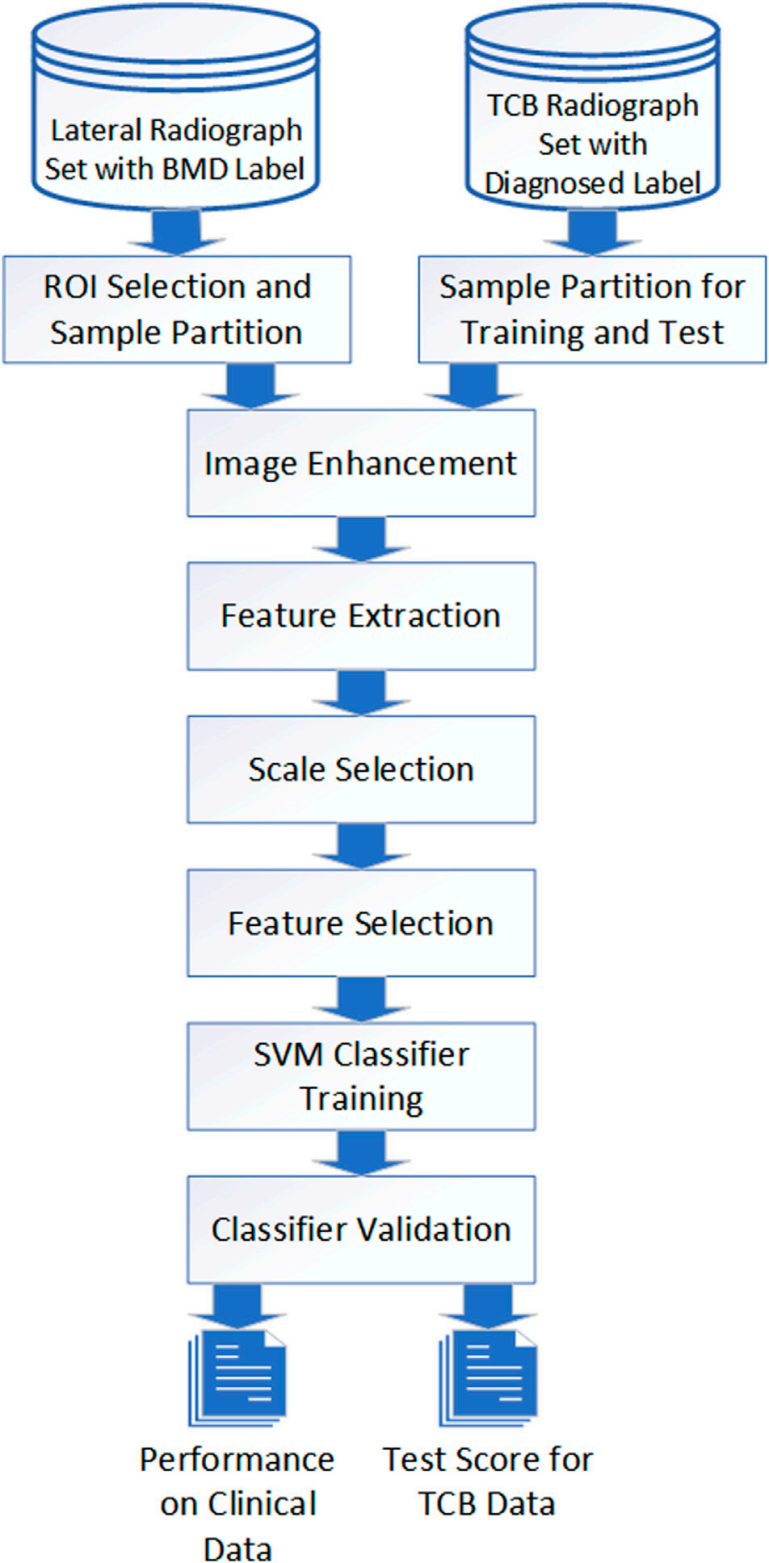
Image process and classification flowchart. Image data from both TCB and clinical went through the same procedure even though different parameters were applied for different cases.

The description of the entire operating procedure from X-rays to diagnosis can be represented by flowchart modules (Figure 1). For different image sources, identical manipulation steps were taken. Those steps included image preparation, enhancement, feature extraction, scale selection, feature selection, SVM training, and validation. However, due to different intrinsic artifacts introduced in image acquisition, differential processing parameters and hyperparameters were applied.

### 2.1 Data acquisition and partition

Two different kinds of radiograph data were collected. One was the lateral radiograph of the lumbar spine from clinical dataset (IRB code: SH9H-2021-T94-2.) of Shanghai Ninth People’s Hospital. Informed consent from patients was exempted due to the retrospective nature of this study. The inclusion criteria were 1) osteopenia and osteoporosis in women aged ≥50 years. They were identified as postmenopausal by radiologists; 2) all subjects had undergone lateral lumbar spine radiography and DXA examinations within 1 month without any other bone treatments in between. According to BMD results (Cosman et al., 2014), all subjects’ radiographs were categorized into three groups which were the normal group (n = 44) with −1 < T-score, the osteopenia group (n = 28) with −2.4 ≤ T-score ≤ −1, and the osteoporosis group (n = 32) with T-score ≤ −2.5.

The other was a lateral calcaneus radiograph from the TCB challenge dataset (Overview, 2022). It was a region of interest (ROI) image set in the trabecular bone of the calcaneus for the evaluation of osteoporosis diagnostic methods. The data used in this study included those of 87 healthy and 87 osteoporotic (with fracture) subjects.

**FIGURE 2:**
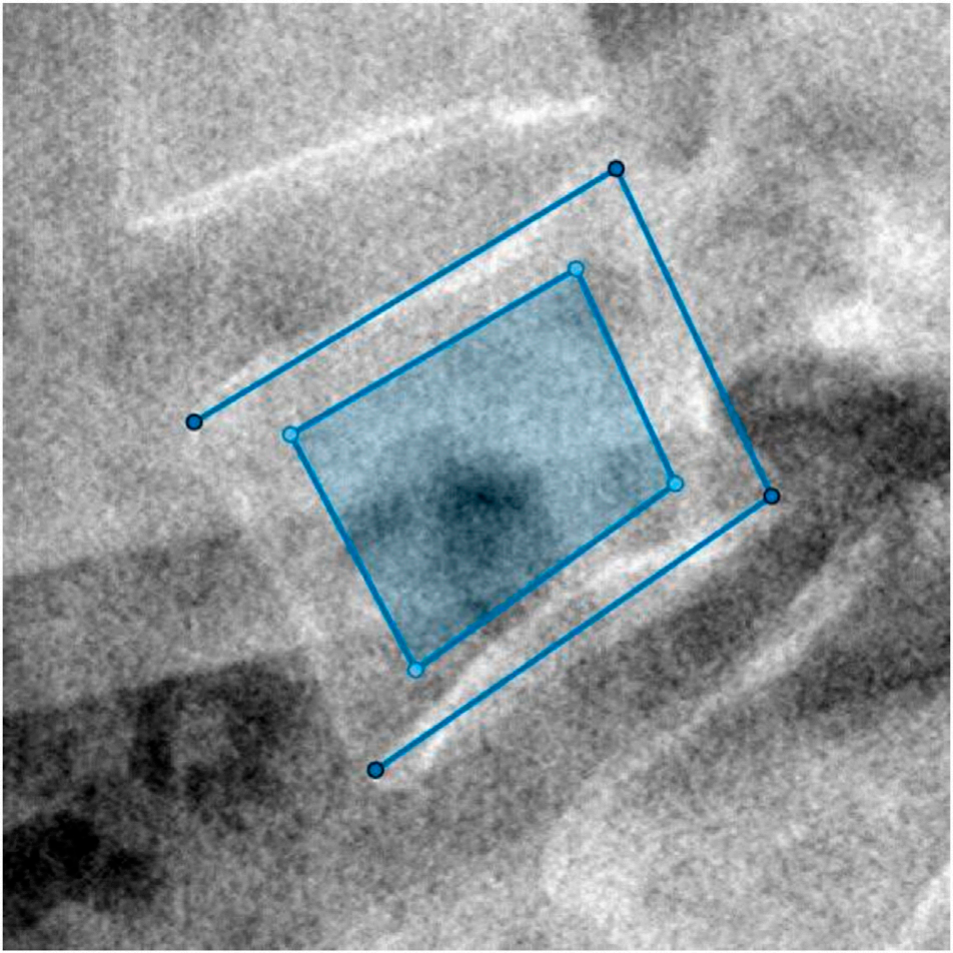
The four vertexes of the outer open quadrilateral selected manually were the four endpoints of endplates’ projection. Then, the dark region cut from the central 2/3 part of the two diagonal of the outer quadrilateral was computed as ROI.

For clinical data, we semi-manually labeled the ROI for the following texture analysis. We utilized MathWorks MATLAB 9.11.0.1769968 to program our labeling tool. Operation steps were as follows: 1) We selected the approximate center of L4 in the original DICOM image and took the point of central focus chosen as the center to crop a sub-view (512*512) of the whole image. 2) In the new cropped lateral view of L4, we manually selected the four corners of vertebral laminae to define the L4 posture. 3) We intercepted the central 2/3 part of the two diagonal. The four breakpoints were taken as the ROI vertexes. The selection and the final ROI of an L4 lateral radiograph are shown in Figure 2.

For the TCB data, 29 subjects were randomly selected from both the control and osteoporosis group. A total of 58 subjects were combined and subjected to the blind test set, in which the health to osteoporosis ratio was 1: 1. For TCB challenges, the ratio of the training set to test set was 2: 1. The mapping between image ID and subject group in the blind test set was saved for classification result assessment.

### 2.2 Image enhancement

**FIGURE 3:**
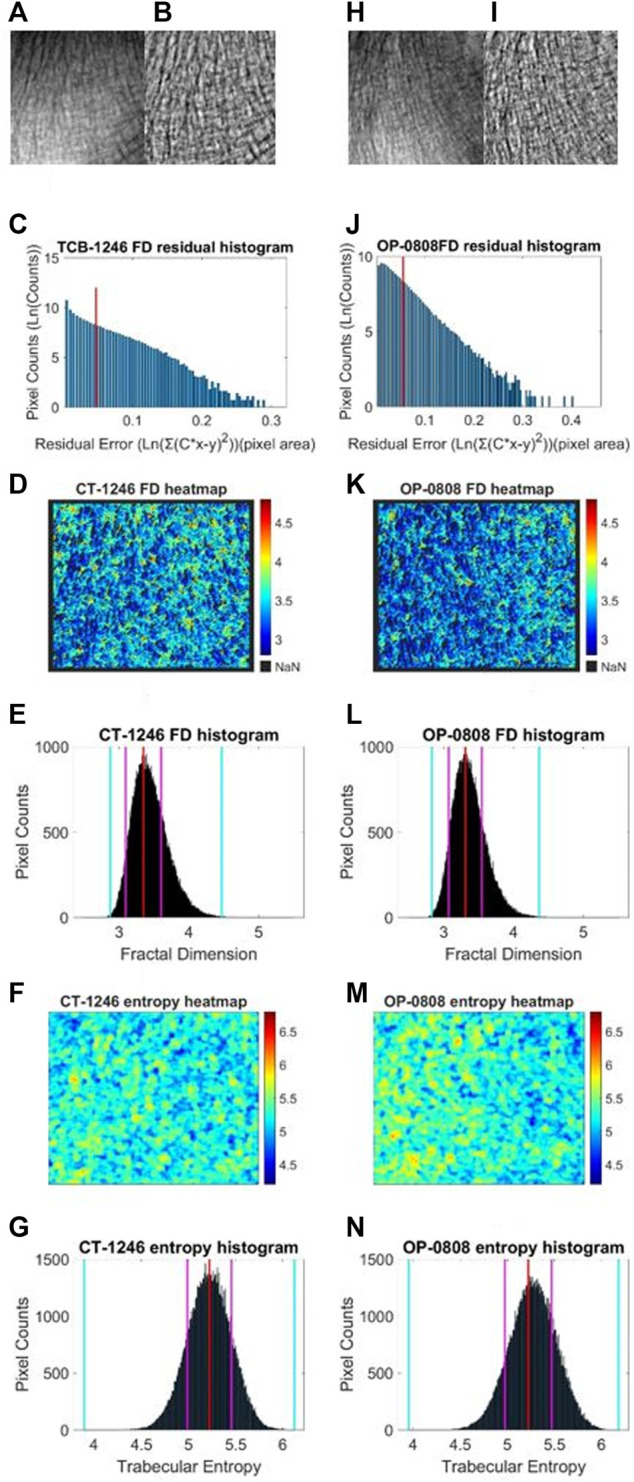
Fractal and entropy features of the TCB dataset. The first column was the analysis of a typical case of the healthy group and the second column was the analysis of a typical case of the osteoporosis group. In the first row, **(A, B)** the raw image from the healthy dataset and the enhanced image after preprocess are shown, respectively. **(H, I)** The same case for the osteoporosis group. In the second row, **(C, J)** log–log fitting residual histograms of pixel-wise FD calculation with binning size 0.005 (ln (gray value)^2^) are shown. The pixels laid on the right side of the red line would be excluded from FD counting. In the third row, **(D, K)** FD heat maps are shown. In the fourth row **(E, L)** FD histograms with binning size 0.005 are shown. For each histogram, the red lines indicate the mode values of FD distributions, two pink lines indicate the ±standard deviation, and two cyan lines indicate the superior and inferior 0.1% quantiles. In the fifth row **(F, M)** entropy heat maps are shown. In the sixth row **(G, N)** entropy histograms with binning size 0.001 are shown; the red lines indicate the mode values of FD distributions, two pink lines indicate the ±standard deviation, and two cyan lines indicate the superior and inferior limits of the whole distribution.

**FIGURE 4:**
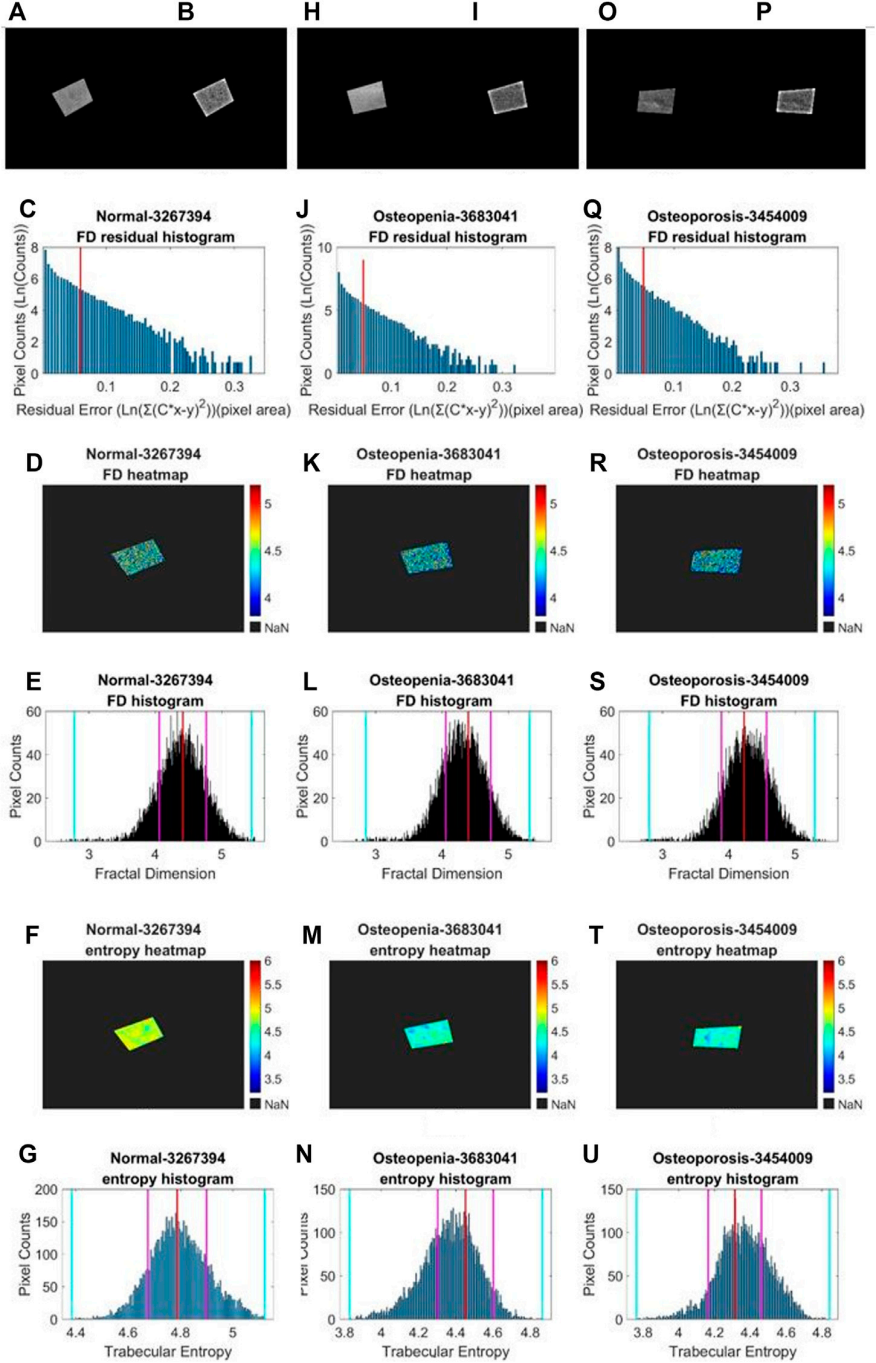
Fractal and entropy features of clinical L4 lateral radiograph dataset. Three columns from **(A, B–G)**, from **(H, I–N),** and from **(O, P–U)** depict typical cases in normal, osteopenia, and osteoporosis individually. In the first row, **(A, B)**, **(H, J)**, **(O, P)** a comparison between the raw image and clean image from each group is shown. In the second row, **(C, J, Q)** log–log fitting residual histograms with binning size 0.005 [ln (gray value)^2^] are shown. In the third row, **(D, K, R)** FD heat maps are shown. In the fourth row, **(E, L, S)** FD histograms with binning sizes of 0.005 are shown. For each histogram, the red lines indicate the mode values of FD distributions, two pink lines indicate the ±standard deviation, and two cyan lines indicate the superior and inferior 0.1% quantiles. In the fifth row, **(F, M, T)** entropy heat maps are shown. In the sixth row **(G, N, U)** entropy histograms with binning size 0.005 are shown; the red lines indicate the mode values of FD distributions, two pink lines indicate the ±standard deviation, and two cyan lines indicate the superior and inferior limits of the whole distribution.

We performed a similar image enhancement operation for both TCB and clinical datasets using different parameters. Due to bone thickness variation in an image or heterogeneous soft tissue projection, X-ray exposure imbalance occurred in a single image of trabecular bone. First, we flattened the ROI with the MATLAB function *imflatfield* with radii 20 for the TCB set and radii 10 for the clinical set. Second, considering the X-ray scatter caused by soft tissue, we used the dehazing method *imreducehaze* to suppress this blurry artifact for bone imaging. The intensity was 0.05 for the TCB set and 0.1 for the clinical set. Third, we adjusted the gray value window width and window level for both TCB and clinical set to make the highest 0.1% pixels saturate and the lowest 0.1% cut-off in each image. Those original images were named as raw images (Figures 3A, H and Figures 4A, H, O), while those enhanced images were named as clean images (Figures 3B, I and 4B, I, P).

**FIGURE 5:**
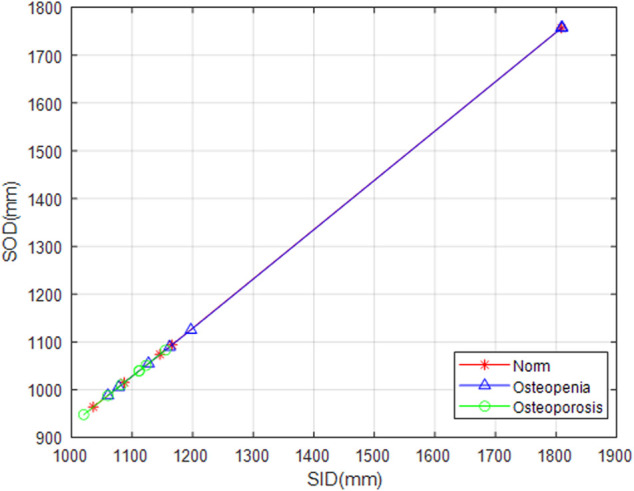
SOD value fitting. Snowflakes, triangles, and circles marked SID against SOD from normal, osteopenia, and osteoporosis groups, respectively. It is a ground breaking uniformization of X-ray projection scale transformation for scale-related feature counting.

Because of clinical images, different projection conditions were applied to different patients, i.e., each radiograph had its respective source-to-image distance (SID) and source-to-object distance (SOD). We unified the image scale diversity. There were two brands of digital radiography (DR), GE Healthcare, and Carestream Healthcare, from which these clinical images were taken. Those DICOM images from the GE Healthcare had both SID and SOD, whereas images from the Carestream Healthcare only had SID. We draw those scatter points from SID to SOD of all GE Healthcare images and linearly interpolate missing values between them. We applied interpolation values for those images with no SOD parameters (Figure 5).

### 2.3 Feature extraction

#### 2.3.1 Pixel-wise fractal dimension (FD)

As Peleg et al.’s (1984) extension on Mandelbrot’s (1994) definition of fractal dimension, when a gray-level image was viewed as a hilly terrain surface, whose height from the ground level is proportional to the image gray value, the area of the fractal surface is displayed according to the power function of measure scale or rather scaled pixel size. Then, the hilly terrain surface was characterized by textural fractal dimension (Creutzberg and Ivanov, 1989). The transformed relationship is shown as follows (Creutzberg and Ivanov, 1989; Enkins et al., 2018):

$$FD=2-\frac{\Delta \ln\left(SA\right)}{\Delta \ln\left(R\right)}.$$ (1)

In Eq. 1, FD means the fractal dimension. SA means the surface area of the image gray value, and it was the mass we wanted to measure in various scales. R stands for the rescaled pixel size. The mechanism of a typical radiographic image mainly is about X-ray attenuation projection on a flat panel detector. All anatomies involving bone and soft tissues that X-ray passed through would be superimposed into the image. The superimposed soft tissue visibly affected the bone texture gray value. So, we employed the blanket method, which was robust against gray-level changes (Lopes and Betrouni, 2009). To further acquire pixel-wise local FD distribution in a single image, we adapted the modified blanket method (MBM) (Enkins et al., 2018) as our final implementation.

We used a mathematical model to disclose the relationship between the surface area and rescaled pixel size rather than changing the whole image resolution. Former studies have shown that we could take pixels as columns and their side area could be equivalent to the image surface area (Peleg et al., 1984; Enkins et al., 2018). Horizontal and vertical gradient were calculated with convolution kernel [1 −1, 0] and [0, −1, 1]^T^, respectively. When we got those two perpendicular direction gradients, it was easy to express the surface area as follows:

$$SA\left(p,r\right)=\underset{{∥p-q∥}_{2}<r}{\sum }\left|G_{x}\left(q\right)\right|+\underset{{∥p-q∥}_{2}<r}{\sum }\left|G_{y}\left(q\right)\right|.$$ (2)

**FIGURE 6:**
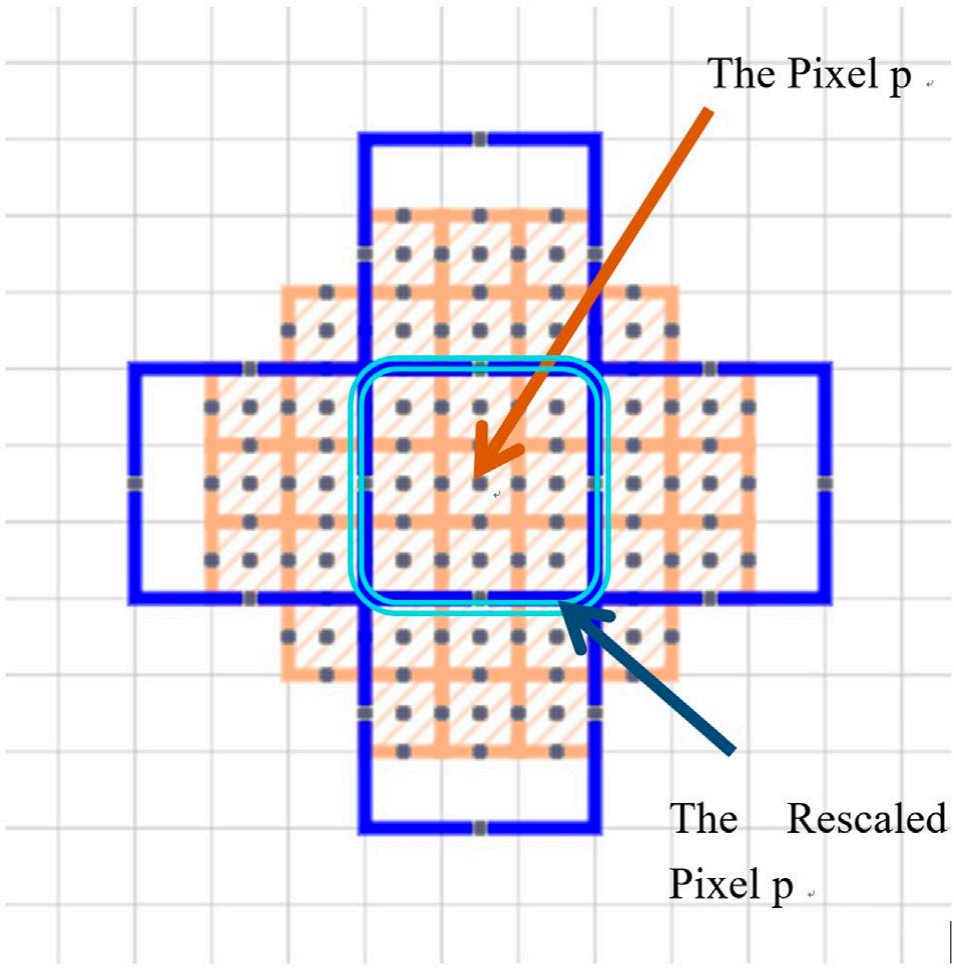
Orange cells and gray cells indicate the original pixels in an image. The center orange cell was the pixel *p*. One example of rescaled pixels centralized at pixel *p* was the innermost blue square. This sample rescaled pixel was composed of nine original physical pixels.

In this equation, $SA\left(p,r\right)$ represents the 3D topological surface area of an image at current scale. The parameter *p* represents the p-th pixel. The parameter r means the radii of a measuring disk in which a local FD value was counted. The physical size of a measuring disk was constant for all rescaled pixel sizes. So, when the rescaled pixel size turned to be bigger, the corresponding r expressed with rescaled pixel would be smaller. $\left|G_{x}\left(q\right)\right|$ and $\left|G_{y}\left(q\right)\right|$ represent the absolute values of the horizontal and vertical gradient at pixel q. Here, q represents any pixel whose distance to the pixel *p* is less than r. As described in the original MBM, the whole image would resemble at a different resolution to rescale the pixel size. For example, all pixels in the same binning patch would have the same pixel value when physical pixel-wise FD was counted. Namely, in Figure 6, all physical pixels inside the pink double-line square region had the same resample value. Nevertheless, in this study, we have made several improvements to the original MBM procedure to obtain a more reliable FD.

**FIGURE 7:**
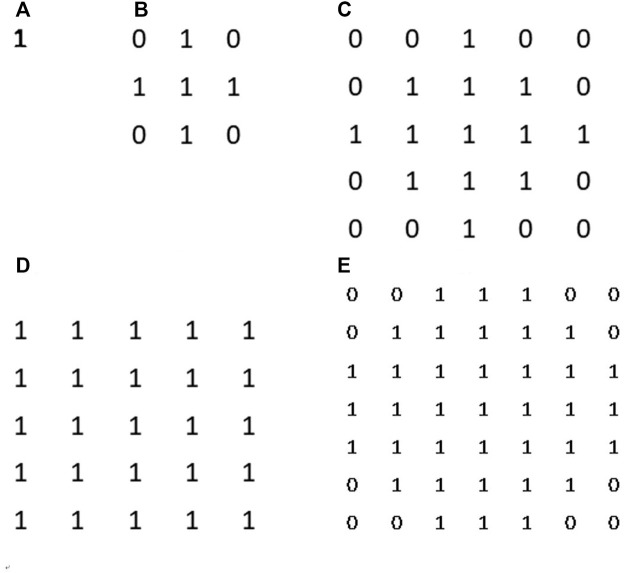
Surface area counting disks. From **(A–D)** they are disk kernel with corresponding radii from 0 to 4. Those disk kernels can help us to restrict a specific neighbourhood around counting pixel for measurement.

First, local surface areas were measured in different binning patches which were centralized at each pixel. When we calculated the local FD of the p-th pixel, the surface of a disc-shaped neighborhood of the p-th pixel, shown in Figure 6, would be measured on different scales. After the physical radii of the neighborhood disk were predefined, a serial of continuously changing rescaled disc radii meant a serial of corresponding pixel binning was taken for a constant physical neighborhood size. In Figure 7, the disk radii changed from 0 to 4. Here, all different sizes of pixel binning centralized at the p-th pixel were taken in the side neighborhood disk. In Figure 6, different color cells distributing around the p-th pixel demonstrated how to centralize at the p-th pixel. The gray cell stood for the physical pixels. The blue cells were rescaled pixels with the binning size 3. Blue cells and orange cells indicated the disc neighborhood of the pixel *p* on a different scale, and they had approximately equal measure regions. So, instead of the original MBM using the whole image multi-resolution, here, different rescaled pixel sizes were all centralized at each pixel and generated pixel-wise FD precisely located at each pixel.

Second, in an image, sporadic pixels which did not characterize fractal significantly would be excluded for FD counting. Due to the influence of imaging noise and superimposed artifacts of radiographs, sporadic pixels’ neighborhoods did not characterize fractal features so well that a few pixels had a relatively high residual value in the residual histograms in Figures 3C, J for the TCB dataset and Figures 4C, J, Q for the clinical dataset. To figure out those pixels, all residuals from the straight-line fitting of the surface area against scaling factors were cached and sorted. The residual distribution could be checked in a log–log histogram with binning size 0.005 [ln (gray value)^2^] in Figures 3C, J, 4C, J, Q. The absolute majority of fitting residuals was close to zero, so we remained with those pixel FDs with 80% of the smallest residuals for further statistics and abolished the other pixels. In Figures 3C, J, 4C, J, Q, those FD values corresponding to the left side of the red line remained, and values on the right side were abolished. We could draw the FD heat maps from these reserved pixel-wise FDs, Figures 3D, K, 4D, K, R, and histogram, Figures 3E, L, 4E, L, S, with binning size 0.005.

Third, more FD distribution features were detected by introducing the FD histogram of the whole image. In Figures 3E, L, 4E, L, S, it is easy to find that the FD distribution shape was basically a single peak but asymmetric in both the control and osteoporosis groups, so we calculated the mode value instead of the mean. Regarding the hackly distribution around the peak, we applied a sliding window (window width = 0.011, moving step = 0.001) to calculate an average value on the histogram and took the maximum as the mode value. In addition, FD standard deviation, FD skewness, FD kurtosis, and the Shannon entropy of FD rescaled to 16-bit unsigned integer were all counted as features.

#### 2.3.2 Differential box-counting (DBC) lacunarity

Although some studies suggested that FD and lacunarity may be correlated in some cases (Smith et al., 1996), lacunarity is by no means redundant. Some patterns are more distinguishable by their lacunarity (Karperien et al., 2011). Especially in our study, the image texture is neither an ideal fractal topological structure nor a disclosed graph, so the lacunarity feature deserves to be tried. The lacunarity of a set implicates the distribution character of mass. A well-accepted approach to calculating lacunarity is as follows:

$$\Lambda \left(r\right)=\frac{\underset{M}{\sum }M^{2}Q\left(M,r\right)}{{\left[\underset{M}{\sum }MQ\left(M,r\right)\right]}^{2}}=\frac{\sigma^{2}\left(r\right)}{\mu^{2}\left(r\right)}+1,$$ (3)

$$where\;Q\left(M,r\right)=\frac{n\left(M,r\right)}{N}.$$

In Eq. 3, $\Lambda \left(r\right)$ is the lacunarity, a function of measure unit r . On the right of the first equal sign, M means the mass of a square area r×r . The probability $Q\left(M,r\right)$ means the number of boxes $n\left(M,r\right)$ with size r and mass M dividing the total number of boxes N in the whole image described in the equation. The mean-square of M divided by its square mean was the lacunarity. The simplified expression is on the right.

In this study, we took the differential box-counting (DBC) algorithm to calculate M at scale unit r (Sarkar and Chaudhuri, 1994; Conci and Campos, 1996; Li et al., 2009). Yet, considering the influence of the initial position of the array of counting boxes, we took an average of all possible initial positions. The average lacunarity, $\bar{\Lambda }$ , is calculated in the following equation. γ implicates the possible initial position, and G equals the number of all possible initial positions, and $\Lambda_{\gamma }$ is the whole image lacunarity Λ at the initial position γ .

$$\bar{\Lambda }=\frac{\sum_{\gamma =1}^{G}\Lambda_{\gamma }}{G}.$$ (4)

#### 2.3.3 Pixel-wise entropy

Shannon entropy quantifies the amount of uncertainty involved in the value of a random variable or the outcome of a random process (Shannon, 2001). Numerous studies validated that focus on measuring the complexity of a chaotic system using information entropy were reliable (Costa and Goldberger, 2015; Humeau-Heurtier, 2016). Nevertheless, most of them were limited in studying unidimensional datasets. Here, we attempted to extend the entropy calculation to conduct a 2D osteoporotic radiography texture analysis.

$$v_{p}=max\left(I_{q}\right)-min\left(I_{q}\right),$$ (5)

$$where\;q\in \left\{q|{∥p-q∥}_{2}\leq r\right\},$$

$$H_{l}=-\sum_{i=1}^{n}P\left(v_{i}\right)\log_{2}P\left(v_{i}\right).$$ (6)

To get robust random value observations, we reused the DBC method in computing lacunarity to count differential value instead of absolute value. First, we set a measuring scale representing a small neighborhood of the p-th pixel in the image ROI with radii r. The difference between the maximum and minimum gray level inside the pixel’s small neighborhood was taken as an observation of the local random measure at that pixel. In Eq. 5, $v_{p}$ is the measure observation at the p-th pixel and q is a pixel inside the small neighborhood of the p-th pixel. Second, we set a big neighborhood, a disc-shaped statistic field, as the local entropy evaluation range, in which observations at every pixel’s small neighborhood were counted to calculate Shannon entropy. When we took the disc-shape statistic field with n pixels inside and moved it as a sliding window to cover the whole image. Then, the local entropy $H_{l}$ was calculated by Eq. 6, in which n is the total number of pixels in the big neighborhood of the p-th pixel and $P\left(v_{i}\right)$ is the probability of the observation $v_{i}$ . In Figures 3F, M, and Figures 4F, M, T the pixel-wise entropy heatmap of the whole image were shown. Furthermore, for each of them, a histogram can be generated in Figures 3G, N and Figures 4G, N, U demostraiting osteoprotsis pathological progress.

After the statistic slide window moved over the whole image, an entropy heat map and its histogram (binning size 0.005) were achieved. The entropy histogram extracted four related features involving mode value, standard deviation, skewness, and kurtosis.

### 2.4 Scale parameter selection

Any classification problem can be solved as a pairwise binary classification problem. In the following section, we only discuss the binary classification performance, in both the TCB challenge case and our clinical bone fragility grading case.

Concerning the scale factor’s impact on the outcome of classification, an optimized scale had to be selected rationally. For FD calculation, we had to decide the finest measure resolution (the smallest scaled pixel size) and the maximum measure range (the biggest scaled pixel size) pixel scale to fit a straight line from the scaled pixel to the surface area. The optimized FD fitting scale was expected to best divide the normal group and the other observed groups. The diversity scale size r in lacunarity calculation, together with the various measure scales and counting scale in entropy statistic, would all lead to a different performance in the classification job.

We employed the Davies–Bouldin index (DBI) (Davies and Bouldin, 1979), a cluster distance index, to evaluate scale factors’ performance in class separations. DBI estimated the class cohesion based on the distance from the feature points in a class to its centroid and the separation based on the distance between centroids. The ratio of cohesion to separation was the DBI value (Arbelaitz et al., 2013). The smaller the DBI, the better the class was separated. The definition formula of the DBI is as follows:

$$DBI\left(C\right)=\frac{1}{K}\underset{c_{k}\in C}{\sum }\max_{c_{l}\in C\setminus c_{k}}\left\{\frac{S\left(c_{k}\right)+S\left(c_{l}\right)}{∥\bar{c_{k}}-\bar{c_{l}}∥}\right\},$$ (7)

$$where\;S\left(c_{k}\right)=\frac{1}{\left|c_{k}\right|}\underset{x_{i}\in c_{k}}{\sum }∥x_{i}-\bar{c_{k}}∥.$$

The parameter C of DBI was the full set of classes to be separated. K is the number of classes in the full set. For this study K = 2. $S\left(c_{k}\right)$ is the average distance from each scatter point to the centroid of K and $x_{i}$ is an n-dimensional feature vector assigned to any point i in class k. $\bar{c_{k}}$ is the centroid of K . ∥∙∥ is the euclidean distance.

*Via* the DBI, we could iterate and clarify which scale size outperformed in this classification job. For two-class classification, we iterated some empirical value scale factors and figured out one or two locally optimized scale sizes for each of those three feature categories. So far, we had 11 features, which are FD mode, FD sigma, FD skewness, FD kurtosis, FD entropy, lacunarity A, lacunarity B, entropy mode, entropy sigma, entropy skewness, and entropy kurtosis with definitive scale size. Lacunarity A and B were the best two distinguishable scale sizes.

### 2.5 Feature selection

We selected six feature dimensions from 11. Feature selection can not only mean the most effective features to classify bone fragility grade but also overcome overfitting and level up generalization performance. *Via* trialing on the TCB dataset, we found six feature dimensions that could provide comparable performance on both the blind test set and the training test. The best six features were selected *via* the popular neighborhood component analysis (NCA) method (Yang et al., 2012). NCA, a non-parametric method, differs from principle component analysis (PCA) and linear discriminant analysis (LDA). PCA was influenced by the scaling of the variables. LDA works only if all class distributions are Gaussian with one single shared covariance (Raghu and Sriraam, 2018). In NCA, the goal was to maximize the prediction accuracy of classification algorithms. On the other hand, in learning feature weights, a regularization parameter λ was introduced to learn feature weights to minimize an objective function that measured the average leave-one-out classification loss over the training data to expect a better generalization performance.

**FIGURE 8:**
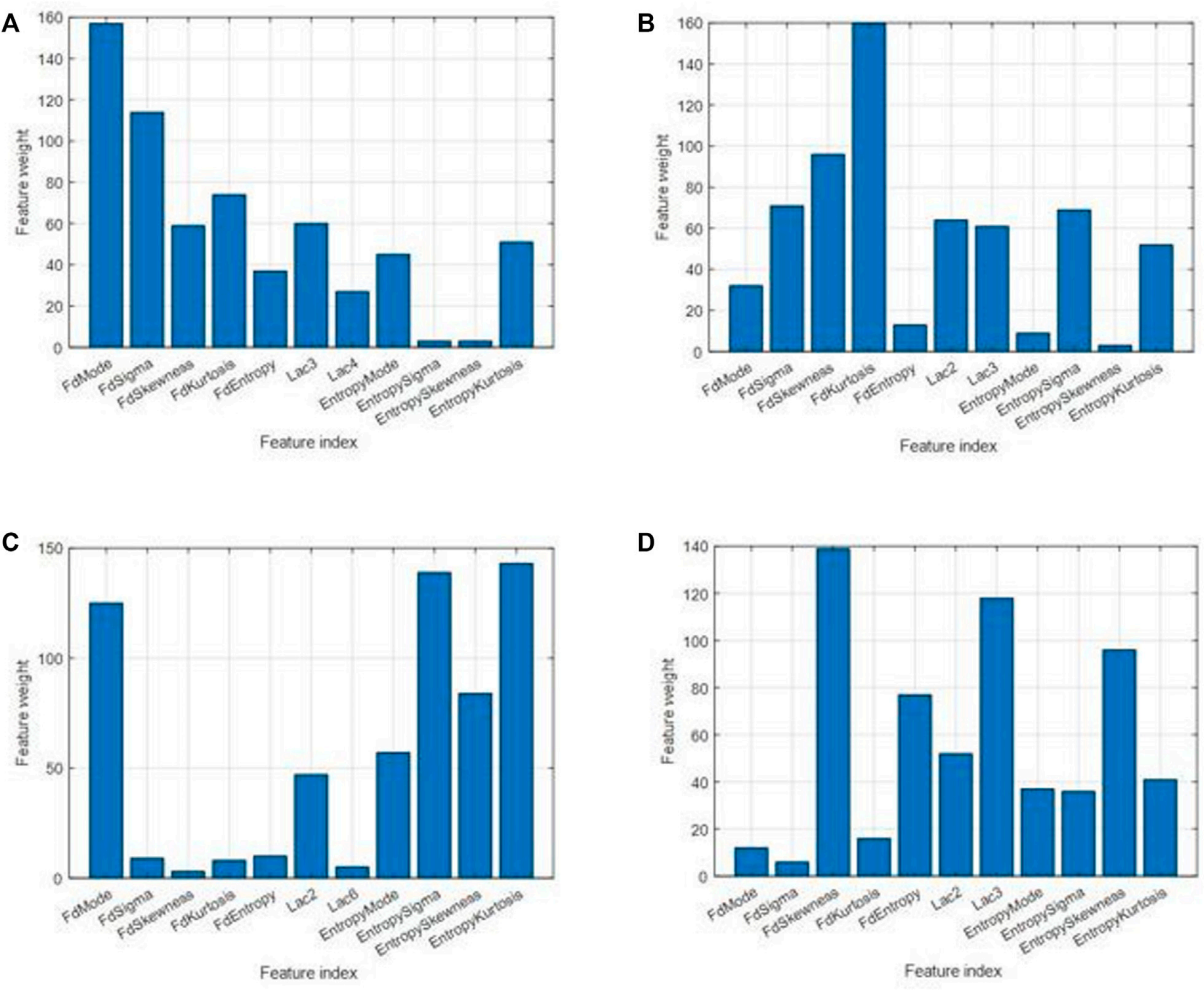
Feature weights for different cases. **(A)** For classification between control and osteoporosis in the TCB dataset. **(B)** For classification between osteopenia and normal in the clinical L4 dataset. **(C)** For classification between osteoporosis and normal in the clinical L4 dataset. **(D)** For classification between osteoporosis and osteopenia in the clinical L4 dataset.

In NCA, 10 steps were taken. i) We partitioned the labeled sample set with 11 dimension features into five folders. In each fold, 4/5 of data were assigned to a training set, and the other 1/5 of data were assigned to a test set; ii) generated a regulation parameter vector, λ , using the MATLAB command λ values = *linespace* (0, 2, 20)/n where n is the number of samples of the whole training set; iii) trained the NCA model with the MATLAB function *fscnca* for each λ using the training set; iv) computed the classification loss for the corresponding test set in the fold using the NCA model and recorded the loss value; v) repeated (iii) for all folds and all λ values; vi) found the λ value that corresponds to the minimum average loss; vii) fitted the NCA model with *fscnca* to all of the data using the best λ value. Meanwhile, we got the fitting loss and feature weights of this round; viii) repeated from (i) to (vii) 60 times and recorded all those fitting losses and corresponding feature weights; ix) sorted the fitting losses and retained the best-fitting ones and their corresponding feature weight rankings. The other half of them was abolished; and x) took each weight ranking as a ballot. Each feature weight ranking had its ballot score [0, 0, 0, 0, 0, 1, 2, 3, 4, 5, and 6] from the least to the most significant and summed up the remaining 30 time ballot scores for each feature. We got the overall ranking for all features. Feature weight bar chats are shown in Figure 8.

### 2.6 SVM training

On the training sets for TCB and clinical L4, the six most significant labeled features of each pair of classes were input into the SVM classification function *fitcsvm*. Configure parameters were listed as follows: “OptimizeHyperparameters,” “all;” “Standardize,” true; “HyperparameterOptimizationOptions,” struct (“AcquisitionFunctionName,” “probability-of-improvement,” UseParallel’, true, “Repartition,” true, “Kfold,” 10, “MaxObjectiveEvaluations,” 800). Considering that the learning process is time consuming, we used the command pair *parpool* and *delete* [*gcp*(“*nocreate*”)] to embrace the *fitcsvm* function, which would be executed in a multi-core CPU in parallel.

### 2.7 Classifier validation

For the TCB blind test, we estimated receiver operating characteristic (ROC), area under the curve (AUC), and Matthews correlation coefficient (MCC) with a 95% confidence interval and 1,000 times bootstrap. In addition, to use the TCB ranking for reference, we also calculated accuracy (Acc), sensitivity (Sn), and specificity (Sp).

For the clinical L4 test set, due to the limitation of sample size on the full dataset, we performed 1,000 times bootstrap. We classified normal, osteopenia, and osteoporosis in pairs. Interval estimates of ROC and AUC in between each pair of them were calculated.

## 3 Results

As hyperparameters, feature counting scales in pixels were optimized by choosing the one with the smallest DBI of pixel-wise FD, global lacunarity, and pixel-wise entropy. For the TCB dataset, the FD radii range was from 1 to 4, and the entropy measure radii and count radii were 1 and 6, respectively. Also, the two optimized lacunarity sizes were 3*3 and 4*4 (Table 1). For the clinical dataset (Table 2), there were three scenarios. In the scenario osteopenia vs*.* normal, the FD radii range was from 1 to 3, the entropy measure radii and count radii were 1 and 9, and the two optimized lacunarity sizes were 2*2 and 3*3, respectively. In the scenario osteoporosis vs*.* normal, the FD radii range was from 0 to 7, the entropy measure radii and count radii were 1 and 6, and the two optimized lacunarity sizes were 2*2 and 6*6, respectively. In osteoporosis vs*.* osteopenia, the radii range was from 1 to 4, the entropy measure radii and count radii were 1 and 7, and the two optimized lacunarity sizes were 2*2 and 3*3, respectively. In tables, those preferred scales were marked in red with a straight underscore and orange with a wave underscore.

**TABLE 1:** Scale size assessment *via* DBI for TCB dataset classification. The red and yellow numbers also in bold stand for the minimum and subminimum DBI of each kind of feature scale parameter list. The corresponding scale parameters of the red and yellow DBI are the optimized ones.

Scale size assessment *via* DBI for TCB classification								
Radii range	1–3	1–4	1–5	1–6	1–7			
FD	4.664	$\underline{3.26}$	3.589	5.361	5.245			
Measure radii, count radii	1,4	1,5	1,6	1,7	1,8	2, 5	2, 6	2, 7
Entropy	7.741	6.535	$\underline{6.428}$	7.035	6.960	10.168	12.968	17.269
Size	2*2	3*3	4*4	5*5	6*6	7*7		
Lacunarity	4.045	$\underline{2.890}$	$\underline{2.939}$	3.151	3.478	3.877		

**TABLE 2:** Scale size assessment *via* DBI for L4 dataset classification. The red and yellow numbers also in bold stand for the minimum and subminimum DBI of each kind of feature scale parameter list. The corresponding scale parameters of the red and yellow DBI are the optimized ones.

Fractal dimension											
Radii	1–3	1–4	1–5	1–6	1–7	1–8	1–9	2–8	0–3	0–4	0–7
Osteopenia vs. normal	$\underline{6.215}$	7.179	7.789	11.006	8.094	7.724	9.552	15.059	8.620	11.044	9.475
Osteoporosis vs. normal	9.524	13.472	11.821	10.014	7.297	7.098	7.455	8.203	10.419	9.178	$\underline{6.774}$
Osteoporosis vs. osteopenia	18.335	$\underline{7.360}$	14.312	13.597	7.364	5.400	9.681	10.425	13.035	15.143	10.153

**Lacunarity**						
Size	2*2	3*3	4*4	5*5	6*6	7*7
Osteopenia vs. normal	$\underline{2.915}$	$\underline{7.826}$	34.919	27.391	23.874	22.148
Osteoporosis vs. normal	$\underline{7.408}$	10.758	9.849	10.310	$\underline{7.548}$	7.900
Osteoporosis vs. osteopenia	$\underline{2.358}$	$\underline{4.367}$	7.229	7.026	5.512	5.564

**Entropy**										
Measure radii, count radii	1,4	1,5	1,6	1,7	1,8	1,9	1,10	1,11	2,6	2,9
Osteopenia vs. normal	19.557	20.941	14.669	11.817	10.407	$\underline{9.910}$	10.471	10.625	10.747	14.478
Osteoporosis vs. normal	6.676	4.697	$\underline{4.225}$	4.289	4.496	4.679	5.060	5.728	5.639	6.352
Osteoporosis vs. osteopenia	7.015	5.204	$\underline{5.117}$	5.242	5.242	5.937	6.123	6.704	7.070	6.753

**FIGURE 9:**
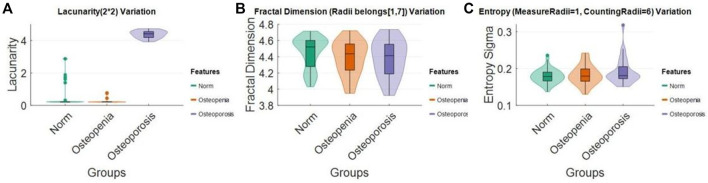
Lacunarity (2*2), FD mode (radii = 1–7), and entropy sigma (measure radii = 1, counting radii = 6) distribution change in **(A)** normal, **(B)** osteopenia, and **(C)** osteoporosis.

Distribution densities of some selected features measured with a fix scale in normal, osteopenia, and osteoporosis were visualized (Figure 9). The lacunarity (2*2) of osteoporosis jumped absolutely out of the range of its value in other cases. However, for the FD mode value and entropy standard deviation, we observed the changing distribution trend, but it was not significant.

The six most effective features were selected, and the effectiveness for classification was ranked by weight (Figure 8). The six most significant features were prioritized: FD mode, FD sigma, FD kurtosis, lacunarity (3*3), FD skewness, and entropy kurtosis for the TCB task. The priority order was FD kurtosis, FD skewness, FD sigma, entropy sigma, lacunarity (2*2), and lacunarity (3*3) for osteopenia vs*.* normal from the clinical L4 dataset. The priority order was entropy kurtosis, entropy sigma, FD mode, entropy skewness, entropy mode, and lacunarity (2*2) for osteoporosis vs*.* normal from the clinical L4 dataset. The priority order was FD skewness, lacunarity (3*3), entropy skewness, FD entropy, lacunarity (2*2), and entropy kurtosis for osteoporosis vs*.* osteopenia from the clinical L4 dataset.

**FIGURE 10:**
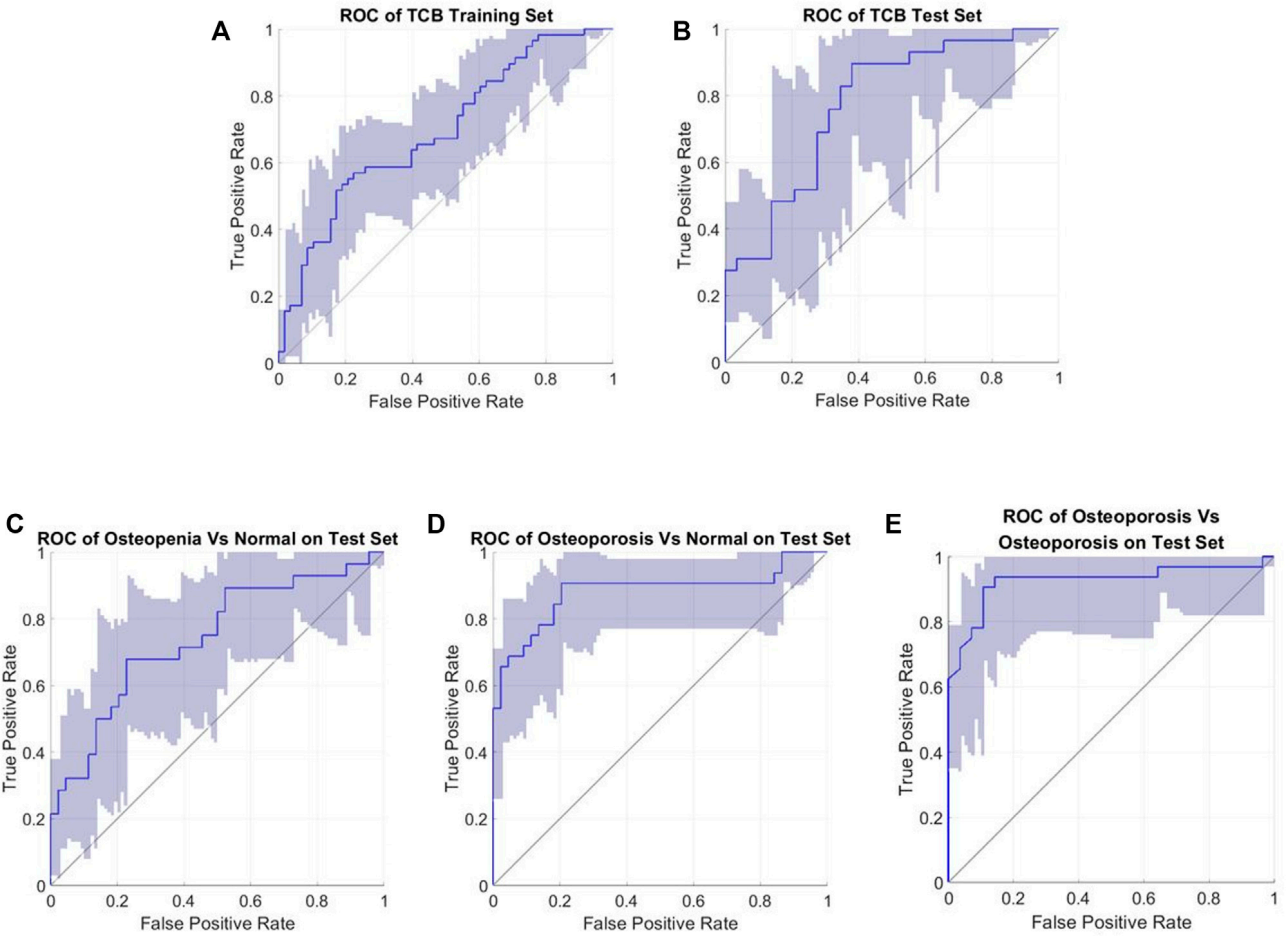
Roc interval estimations (confidence interval 95%). **(A)** TCB training set; **(B)** TCB test set; **(C)** test for osteopenia vs. normal; **(D)** test for osteoporosis vs. normal; and **(E)** test for osteoporosis vs. osteopenia.

All related ROC interval estimations on the test set are depicted in Figure 10. The interval estimation classification on L4 is listed in Table 4. For osteoporosis diagnosis, the AUC, Acc, and Sn (recall) were 0.851 (95% CI: 0.730–0.922), 0.813(95% CI: 0.718–0.878), and 0.936 (95% CI: 0.826–1), respectively. For osteopenia diagnosis, the AUC, Acc, and Sn were 0.721 (95% CI: 0.578–0.824), 0.675 (95% CI: 0.563–0.772), and 0.774 (95% CI: 0.635–0.878), respectively. For osteoporosis diagnosis from osteopenia, the AUC, Acc, and Sn were 0.935(95% CI: 0.830–0.968), 0.928 (95% CI: 0.863–0.963), and 0.910 (95% CI: 0.746–1), respectively.

Our TCB training set and test set were repartitioned, but the performance was comparable with the other feature-based and SVM classifiers within the TCB ranking (Table 3). For the training dataset, the AUC, Acc, and Sn were 0.694 (95% CI: 0.592–0.778), 0.663 (95% CI: 0.569–0.741), and 0.742 (95% CI: 0.607–0.839), respectively. For the blind test set the AUC, Acc, and Sn were 0.767 (95% CI: 0.629–0.879), 0.672 (95% CI: 0.545–0.793), and 0.790 (95% CI: 0.621–0.923), respectively. Here, the Acc was slightly higher than that of the method from the work of Florian et al.

**TABLE 3:** Presented classifier with the Acc marked in red was compared with the TCB ranking.

Rank	Author	TP	FP	TN	FN	Sn	Sp	Acc	F1 score
	Presented classifier^a^	16	6	23	13	0.552	0.793	$\underline{0.672}$	0.628
1	Yger (2014)	19	10	19	10	0.655	0.655	0.655	0.655
2	Palanivel et al. (2020)	17	12	17	12	0.586	0.586	0.586	0.586
3	Christoph et al. (Ranking, 2022)	24	22	7	5	0.828	0.241	0.535	0.640
4	Jerome et al. (Ranking, 2022)	21	17	12	8	0.724	0.414	0.569	0.62

^a^ The whole image dataset was repartitioned into a train set and blind test set with the prescribed ratio of 2:1.

## 4 Discussion

Unlike most of the previously reported classifiers (Zheng and Makrogiannis, 2016; Oulhaj et al., 2017; Palanivel et al., 2020; Omiotek et al., 2021), our study exhibited that a novel classifier synthesizing pixel-wise fractal, entropy, and global lacunarity can distinguish three levels of bone mass loss of lumbar spine into normal, osteopenia, and osteoporosis. In addition, it also showed high generalization on osteoporosis diagnosis using calcaneus according to lateral radiographs, which are both routine physical examinations in a broader sense. For a typical DXA examination focus, such as the vertebral spine, or non-DXA examination, such as the calcaneus, the proposed classifier can be successfully applied. The performance of the proposed classifier was reliable, indicating whatever the T-score or the fractured state as the golden standard. In all aforementioned scenarios, Acc is higher than 0.66 and Sn is higher than 0.74. This method can be a promising tool to diagnose osteopenia and osteoporosis on X-ray images for further clinical application.

Several features trended from normal health to osteopenia and osteoporosis. We can try to partially explain the physio-pathological change process of a bone trabecular structure. From Figure 9A, lacunarity drastically rose in the process of pathological changes from normal to osteoporosis. It means the image intensity turns more heterogeneous in the whole image. This phenomenon suggests some lacunarity measure patches of bone degeneration far different from the other groups. In Figure 9B, the FD population distribution of each group mildly declined. This trend could be checked with median values and lower bounds. Lower FD means the bone mass loss happened on a considerable scale range. From Figure 9C, we found that each group’s pixel-wise entropy population’s standard deviation sigma slightly increased. Median values and upper bound depicted the increase. From Figures 3G, N, 4G, N, U, we knew the pixel-wise entropy distribution was unimodal. According to information theory (Chung et al., 2017), the sigma of unimodal distribution indicates the population entropy. So, in other words, the worse the osteoporosis, the higher the global entropy, which is generally following the entropy law, also named the second law of thermodynamics (Schneider and Kay, 1994). The osteoporotic pathogenesis was unprecedentedly depicted through the 2D projection entropy. The entropy could be taken into account as a new osteoporosis biomarker.

The classifier performance was comparable to other latest reports. The state-of-the-art DL-based classification models held an average accuracy of 90.1% ranging from 70.0% to 98.9% and a mean AUC of 0.90 ranging from 0.74 to 1.00 (Zhang et al., 2020) for only osteoporosis diagnosis from healthy subjects. As shown in Table 4, roughly speaking, from the performance point of view, our feature-based model matched with those DL-based models. For osteopenia diagnosis, as we know, the latest DL-based model (Zhang et al., 2020) acquired an AUC of 0.629 with 95% CI: (0.545, 0.707) and sensitivity of 50% with 95% CI: (32.4, 67.6). As in Table 4, our performance, with an AUC of 0.741 with 95% CI: (0.598, 0.844) and sensitivity of 79.4% with 95% CI: (0.655, 0.898), was comparable. High-sensitivity results in a low fraction of false-negative classifications, in other words, a low missed diagnosis rate. Apart from the aforementioned, we also classified osteoporosis from osteopenia. The AUC and Acc are even better than the osteoporosis diagnosis in Table 4. In summary, it is possible to screen osteoporosis from the lateral radiograph of L4 with interpretable features matched with DL-based classifiers.

**TABLE 4:** Classification performance of L4 on the clinical dataset.

Scenario	AUC	Accuracy	Precision	Sensitivity	Specificity	F1 score	MCC
	(95% CI)	(95% CI)	(95% CI)	(95% CI)	(95% CI)	(95% CI)	(95% CI)
Osteopenia vs. normal	0.721	0.675	0.732	0.774	0.537	0.757	0.342
	(0.578, 0.824)	(0.563, 0.772)	(0.592, 0.833)	(0.635, 0.878)	(0.351, 0.720)	(0.659, 0.855)	(0.114, 0.547)
Osteoporosis vs. normal	0.851	0.813	0.807	0.936	0.690	0.874	0.681
	(0.730, 0.922)	(0.718, 0.878)	(0.679, 0.898)	(0.826, 1)	(0.483, 0.829)	(0.783, 0.932)	(0.501, 0.811)
Osteoporosis vs. osteopenia	0.935	0.928	0.964	0.910	0.967	0.943	0.899
	(0.830, 0.968)	(0.863, 0.963)	(0.810, 1)	(0.746, 1)	(0.829, 1)	(0.849, 0.986)	(0.699, 0.967)

The TCB challenge dataset showing characteristic texture had been held since 2014, and here, it was also utilized to show the high generalization of our classifier. The present TCB challenge requires participants to use the SVM classifier, and so, only feature-based methods were taken into account in the ranking list according to their accuracy. In this dataset, the number of osteoporosis cases is identical to the number of healthy cases, and the case ratio for train and test was 2 to 1. To the best of our knowledge, counting follow-up studies are listed in Table 3. On the TCB challenge, no report has a better score than ours yet, regardless of our random dataset repartition. The latest hit list models (Palanivel et al., 2019; 2020) have paid attention to the heterogeneity of the texture and combined multifractal features with lacunarity to gain improvement. However, at the early stages of osteoporosis, bone loss happens at individual trabecular plates (Cosman et al., 2014; Golob and Laya, 2015), so our pixel-wise feature counting has the potential for early diagnosis of osteoporosis.

The proposed image preprocess is advantageous to the following texture feature extraction. The fractal dimension, lacunarity, and entropy are scale sensitive to all three feature categories. They all depicted one facet of trabecular structure changes, whereas the source-to-image distance (SID) and the source-to-object distance (SOD) were not uniformed among clinical radiographs. We carried out imaging scale (pixel size) normalization by SOD vs. SID line fitting for radiographs. Concerning the curved shape of human bones whether lumbar spine or calcaneus, the radiography image intensity was naturally biased. Especially for lateral radiographs of the lumbar spine, there was the thickness of fat, organs, and even clothes having an overlay projection on the image. To limit those negative impacts, we compensated for the bias with Gaussian smoothing. Due to the influence of the thickest soft tissue on the human body, lateral radiographs of the lumbar spine were a blurred with scatters. We utilized the dehaze algorithm to enhance the detailed information on bone texture.

A robust measure, the image surface area, was adopted to overcome the superposition imaging of other human organs and tissues rather than the image intensity analyzed by previous studies (Zheng and Makrogiannis, 2016; Hassouni et al., 2017). Lateral radiographs of the lumbar spine are widely available with low cost. However, subjects’ abdominal organs, cavities, and intestinal gas even waist belts could be all projected in radiographs. Image surface area counting was practiced to overcome those complex impacts (Lopes and Betrouni, 2009; Areeckal et al., 2018b). Fractal dimension features were taken with the local surface area as a measure. Lacunarity and local entropy features were taken based on intensity difference which was a derivative method of image surface measure.

In our study, the proposed model capacity was well restrained to only six feature dimensions not only for better generalization but also as a good foundation for further regressive analysis. In the field of ML, a principle of parsimony (Vapnik, 1999; Vapnik and Chervonenkis, 2015) named Occam’s razor is widely acknowledged; among competing hypotheses that explain known observations equally well, we should choose the “simplest” one. Furthermore, the proposed hyperparameter optimization, feature selection, and classifier training process arguably follow the concept of ensemble learning which had the property of improving generalization (Hansen and Salamon, 1990). Compared with the latest studies for osteoporotic diagnosis, a convolutional neural network (CNN) (Yamamoto et al., 2020; Zhang et al., 2020)-based model had dozens or even hundreds of non-explanatory and abstract features, and real feature-based models (Oulhaj et al., 2017; Palanivel et al., 2020) also collected dozens of feature dimensions to obtain an ideal performance. What we gained from restrained feature dimensions was a better generalization. The AUC of 0.767 with 95% CI: (0.622, 0.874) of the TCB test dataset was not worse than the AUC of 0.694 with 95% CI: (0.591, 0.778) in the training dataset.

There are still several limitations of this study. First, as the golden standard, the BMD value to diagnose osteoporosis is still questionable and challenged by bone histomorphometry studies (Wainwright et al., 2005; Greenwood et al., 2018), so a high-resolution CT exam is suggested as a powerful complement to the DXA exam for osteoporotic diagnosis (Greenwood et al., 2018). Second, the labeled sample size is restricted to a bit more than one hundred. The data scale is not sufficient and diverse to bring this method to real clinical trials, especially for a triple-classification problem, in which osteopenia and osteoporosis are both analyzed. Third, in this study, for vertebral spine radiography analysis, only L4 was analyzed, which is similar to a recent study (Lee et al., 2020). Nevertheless, a DXA scan is from L1 to L4. A corresponding skeleton feature extraction could be a better way to obtain classification results matching T-scores.

This method exhibited the capacity of resisting disturbance on lateral spine radiographs and high generalization on the calcaneus dataset. Interpretable pixel-wise features not only helped to better understand osteoporosis on radiographs but also shed new light on computer-aid diagnosis for osteopenia and osteoporosis.

## Data Availability

The original contributions presented in the study are included in the article/Supplementary Material, further inquiries can be directed to the corresponding authors.
